# Commentary: Cortical activity in the null space: permitting preparation without movement

**DOI:** 10.3389/fnins.2017.00502

**Published:** 2017-09-13

**Authors:** Mikhail A. Lebedev

**Affiliations:** Department of Neurobiology, Duke University Durham, NC, United States

**Keywords:** neuronal ensemble, neuronal tuning, multitasking, subspace, motor cortex, premotor cortex, population vector

Kaufman et al. recently proposed a hypothesis of how cortical neuronal ensembles prepare movements without initiating them prematurely (Kaufman et al., 2014). Although novel and potentially paradigm-shifting, their model appears to contradict some of the previously reported results. Here I discuss several possible reasons for this contradiction.

Kaufman et al. recorded from neuronal populations in dorsal premotor cortex (PMd) and primary motor cortex (M1), in monkeys performing center-out arm reaching movements with straight and curved trajectories. The experimental task incorporated a delay period during which monkeys could see the target but were required to withhold movement until a trigger stimulus (Figure 1A). Kaufman et al. asked how it was possible that M1 and PMd, known to project to the spinal cord and to each other (Dum and Strick, 2002), modulated their activity in in a time- and direction-dependent manner during the delay but did not induce EMG responses. While the standard explanation has been that delay-period cortical activity is a subthreshold version of movement activity (Tanji and Evarts, 1976; Weinrich and Wise, 1982; Alexander and Crutcher, 1990; Riehle and Requin, 1995; Prut and Fetz, 1999), Kaufman et al. proposed an alternative explanation. They asserted that delay-period cortical modulations were confined to a null space with respect to the linear transformation that mapped neuronal activity into movements.

**Figure 1:**
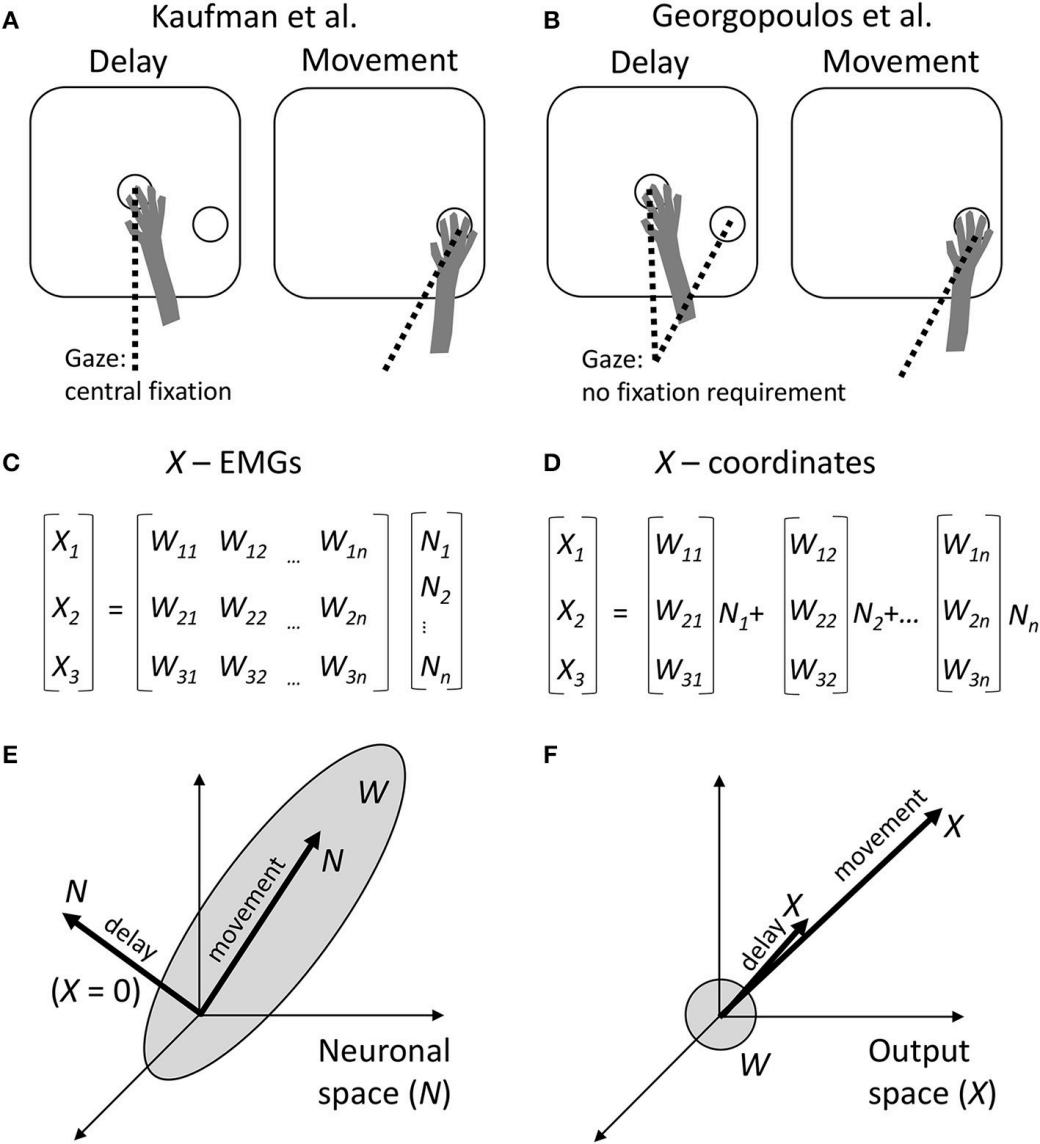
Comparison between the approaches of Kaufman et al. and Georgopoulos et al. **(A)** Schematics of the experimental task of Kaufman et al. During the delay period, a monkey held the arm at the central position and was required to fixate that position with the eyes. The target of movement was visible, but the monkey had to withhold arm and eye movements. After the central fixation point disappeared, the monkey reached toward the target and was allowed to break eye fixation. **(B)** Experimental task of Georgopoulos et al. The task did not constrain eye movements. Possibly, during the delay the monkeys fixated either the central location or the target, and during the movement they fixated the target. **(C)** Conversion of neuronal activity into the outputs by Kaufman et al. A linear transformation *W* is applied to neuronal rates *N* to produce the outputs *X*. **(D)** Georgopoulos' population-vector approach. Although the same type of linear transformation is used, it is expressed somewhat differently as a sum of neuronal vectors [*W*_*1i*_ *W*_*2i*_ *W*_*3i*_] weighted by neuronal rates *N*_*i*_. **(E)** The result of Kaufman et al. The transformation *W* defines a subspace in the neuronal space, called output-potent space. During the movement period, neuronal population activity *N* resides in this subspace. However, during the delay, *N* resides in the null space, so *X* is close to zero. **(F)** Georgopoulos' result. The neuronal vectors point in various directions in the output space, defining roughly a sphere. The population vector points in the direction of movement during both the movement period and delay period of the task. This disagrees with Kaufman's zero output during the delay.

Mathematically, this transformation is expressed by the equation (Figure 1C):

(1) $$X_{i}(t)=\Sigma W_{ij}\;N_{j}(t)$$

where *t* is time, *N*_*j*_*(t)* is the j-th neural input, *X*_*i*_*(t)* is the i-th motor output, and *W*_*ij*_ is the matrix of linear regression weights. *N*_*j*_*(t)* could correspond to single neurons, but in Kaufman's analysis they are principal components derived from the neuronal population activity. (Representation as principal components was needed to reduce data dimensionality and make inputs *N*_*j*_*(t)* uncorrelated). For the conversion of M1 and PMd activity to arm EMGs, *X(t)* corresponds to the EMGs; and for the conversion of PMd activity into M1 activity, *X(t)* corresponds to M1 activity.

Kaufman et al. computed *W*_*ij*_ for the movement period of the task. Next, they applied this transformation to the delay period and discovered that the values of *X*_*i*_*(t)* were very small. An output equal to zero defines the null space of matrix *W*:

(2) $$\Sigma W_{ij}\;N_{j}(t)=0$$

The null-space and output-potent portions of cortical activity were quantified as the variance of the projection of *N* to the null space and row space of *W*, respectively. This analysis confirmed that the delay-period activity resided mostly in the null space (Figure 1E).

While this idea appears quite interesting, the linear transformation defined by Equation (1) and Figure 1C is not new. Indeed, this transformation matches the classical population-vector model proposed by Georgopoulos et al., where population output is represented by a sum of individual neuronal vectors (Georgopoulos et al., 1986; Figures 1D,F). In the Georgopoulos notation, index *i* corresponds to Cartesian coordinates x, y, and z; [*W*_*1j*_ *W*_*2j*_ *W*_*3j*_] is the vector for the j-th neuron; and [*X*_*1*_*(t) X*_*2*_*(t) X*_*3*_*(t)*] is the population vector. Georgopoulos et al. proposed that the population vector tracks movement trajectory (Georgopoulos et al., 1986) and/or mental transformation of a spatial cue into movement direction (Georgopoulos et al., 1989a), but did not apply their analysis to decoding EMGs from neuronal activity.

Given the correspondence between the two approaches, it is somewhat surprising that Georgopoulos et al. reached a very different conclusion based on their analysis of M1 data collected under experimental conditions (Figure 1B) that were very similar to the instructed-delay task of Kaufman et al. (Georgopoulos et al., 1989b; Smyrnis et al., 1992). Georgopoulos et al. reported that “the population vector during the delay period pointed in the direction of movement that was to be made later.” In other words, a portion of the delay-period neuronal activity did lay in the output-potent space of *W*, although it is not clear how substantial that portion was compared to the null-space activity (Figure 1F). (Kaufman's analysis should be run on Georgopoulos' data to clarify this issue).

What could be the reasons for the difference between the conclusions of the two groups? A comparison of Kaufman's and Georgopoulos' tasks reveals a distinction: Kaufman's monkeys maintained central eye fixation during the delay (Figure 1A), whereas Georgopoulos' monkeys did not do so (Figure 1B). This is an important difference because gaze angle significantly influences neuronal activity in both PMd (Boussaoud et al., 1998; Lebedev and Wise, 2001) and M1 (Baker et al., 1999). The effect of eye position on neuronal tuning to target location has been described in terms of an eye-centered coordinate frame (Batista et al., 1999). Furthermore, it has been shown that M1 and PMd process multiple variables during instructed delays rather than representing solely motor preparation. For example, PMd neurons represent orientation of selective spatial attention unrelated to the location of the motor target (Lebedev and Wise, 2001). In behavioral tasks that require reorienting attention and/or sensory-motor transformations, the population vector rotates from the initial focus of attention to the location of the target (Georgopoulos et al., 1989a; Wise et al., 1996). Additionally, delay-period activity of M1 and PMd neurons represents the elapsed time (Renoult et al., 2006; Lebedev et al., 2008).

Given this previous literature, it is reasonable to suggest that both Georgopoulos' and Kaufman's results were impacted by extra behavioral variables, such as eye position and orientation of spatial attention. Specifically, the central eye fixation requirement possibly affected directional tuning in Kaufman's experiments. When a monkey knows target location but should continue central fixation, a misalignment is introduced between the gaze angle, orientation of spatial attention and the prepared movement. Additionally, the presence of two foci of attention, the central fixation point and the target, could have resulted in relative-location encoding (Olson, 2003). All these factors could contribute to a rotation of the directional tuning characteristics away from the ones defined by the movement period. Somewhat similar rotations of neuronal preferred direction have been found for a variety of motor behaviors that incorporated contextually different task periods (Sergio and Kalaska, 1998; Johnson et al., 1999; Lebedev et al., 2005; Churchland and Shenoy, 2007). In Kaufman's case, the null space may have corresponded to an eye-centered coordinate frame and shifts in spatial attention rather than being a mechanism that suppresses premature movements.

An alternative possibility is that Kaufman et al. were right, but Georgopoulos et al. overlooked the contamination of their results by the eye position effects. In this view, target fixation by the eyes during both the delay and movement periods could have resulted in the similarity of neuronal tuning characteristics for these periods. Consequently, Georgopoulos' population vector would have pointed to the target both before and after the movement, reflecting the tendency for the eyes to fixate the target. By contrast, Kaufman's experimental design allowed to measure *W* for the arm movements more cleanly, without the confounding effect of target fixation. In this scenario, there are two possibilities for cortical representation of eye movements. The first possibility is that Kaufman's rules apply to the eye movements the same way they apply to the arm movements: oculomotor preparatory activity resides in the null space during the delay and then shifts into the output-potent space during the saccade. If this were true, literature on oculomotor tasks would have reported differences in neuronal preferred directions between the preparatory and saccade periods. Instead, Chen and Wise compared delay-period and pre-saccadic activity in supplementary eye field, and found that neuronal preferred directions were positively correlated for these periods (Chen and Wise, 1996). Furthermore, Kaufman et al. seem themselves to favor the second possibility, that eye movements are controlled differently from the arm movements. They mention that oculomotor control may employ a gate mechanism to inhibit saccades, with burst neurons and omnipause neurons in the brainstem (Evinger et al., 1982).

An additional point that I would like to make is about the interpretation of the linear transformation defined by equation 1. Kaufman et al. proposed that the regression weights, *W*, correspond to the actual connectivity between the cortex and spinal motoneurons, or between PMd and M1. Alternatively, the regression offers an artificial representation unrelated to any real connectivity. While linear regression utilizes correlations between the inputs and outputs to compute the weights and produce the best fit, it is well-known that correlation is not a proof of causality. Correlations between cortical neurons and EMGs could result from a common input or a common processing mechanism with multiple feedforward and feedback loops, instead of simple, unidirectional connectivity. For example, M1 neurons correlate with arm EMGs even when spike-triggered averaging fails to reveal short-latency connectivity (Zhuang et al., 2014). One could argue that the validity of a linear model could be tested using a cross-validation procedure, where trials or task conditions are left out. Although such tests have shows that the model generalizes to a slightly different motor condition (Santucci et al., 2005; Kaufman et al., 2014), common-input mechanism still remains a possibility. Linear regressions that assign weights to neurons to generate a desired output are commonly utilized in brain-machine interfaces (BMIs) (Humphrey et al., 1970; Wessberg et al., 2000; Lebedev et al., 2005; Lebedev, 2014), but the goal is different there. BMIs make use of the correlation between the inputs and outputs to optimize the decoding, no matter what neuronal mechanism underlies the correlation. BMI decoders typically would not generalize to a very different motor task, such as switching from forward walking to backward walking (Fitzsimmons et al., 2009), which indicates that such decoders do not capture the true causation. Overall, it is not surprising that a linear algorithm can be trained to transform cortical population activity into EMGs, or PMd activity into M1 activity, and that this algorithm would not generate any meaningful output when applied to a different task period. Yet, it is more surprising that the null space generates a more meaningful (or at least higher-amplitude) output.

In conclusion, it appears that future applications of the approach proposed by Kaufman et al. could benefit from the comparison with the results obtained with the traditional methods for assessing neuronal tuning properties, and considering the contribution of behavioral variables different from merely arm movement parameters. Some of the controversies between Kaufman et al. and Georgopoulos et al. could be addressed by more thoroughly testing the effect of eye position, for example fixation could be enforced universally, even during the reach, so that the eye-centered coding components of the neurons would remain fixed between the two epochs. At the modeling level, it may be useful to build a model that incorporates eye position as an additional output. Yet, one should be cautious when interpreting the modeling results in terms of neuronal connectivity. To avoid misleading population results, going back to single neurons and assessing their tuning properties individually (as in Georgopoulos approach) could be useful. Finally, when an interesting population effect is discovered, it would be helpful to examine whether this effect is a mere reflection of the features present in each individual neuron or a unique ensemble phenomenon invisible at the level of single neurons.

## Author contributions

The author confirms being the sole contributor of this work and approved it for publication.

### Conflict of interest statement

The author declares that the research was conducted in the absence of any commercial or financial relationships that could be construed as a potential conflict of interest.
